# Supplementary material for: Mycobacterial cell division arrest and smooth‐to‐rough envelope transition using CRISPRi‐mediated genetic repression systems
Source: FEBS Open Bio. 2025 Nov 30;16(7):1271–92. doi: 10.1002/2211-5463.70172 (PMC13327033; doi:10.1002/2211-5463.70172)
Supplement: Supplementary file 1 — Table S1. List of the primers used in this study to construct Mycobacterium abscessus CRISPRi hypomorphs. Table S2. List of the plasmids used in this study. [file FEB4-16-1271-s001.docx]

**Supporting Information**

Additional information may be found online in the Supporting Information section at the end of the article

**Mycobacterial cell division arrest and smooth-to-rough envelope transition using CRISPRi-mediated genetic repression systems**

Vanessa Point^1#^, Wafaa Achache^1,2#^, Janïs Laudouze^1#^, Eliana Sepulveda Ramos^1#^, Mickaël Maziero^1#^, Céline Crauste^3^, Stéphane Canaan^1^ & Pierre Santucci^1^*

^1^ Aix Marseille Univ, CNRS, LISM, IMM FR3479, IM2B, Marseille, France

^2^ IHU Méditerranée Infection, Aix-Marseille Univ., France.

^3^ IBMM, Univ Montpellier, CNRS, ENSCM, 34000 Montpellier, France.

^#^ Contributed equally as co-first authors

* Correspondence address to Pierre Santucci, [psantucci@imm.cnrs.fr](mailto:psantucci@imm.cnrs.fr)

ORCID <https://orcid.org/0000-0002-6291-3425>

**Table S1. List of the primers used in this study to construct *M. abscessus* CRISPRi hypomorphs**

| **Number** | **Name** | **Restriction site**  (if applicable) | **Sequence 5'-3'** | **Vector** (if applicable) |
| --- | --- | --- | --- | --- |
| #P1 | CRISPRi-pIRL-Sequencing | - | TTCCTGTGAAGAGCCATTGATAATG | - |
| #P2 | pIRL_*mmpL3*_MAB4508_sg1_Top | BsmBI-Cut | GGGAGCCATTCGGCCCCACCAGGCGAAC | pEAR46 |
| #P3 | pIRL_*mmpL3*_MAB4508_sg1_Bottom | BsmBI-Cut | AAACGTTCGCCTGGTGGGGCCGAATGGC | pEAR46 |
| #P4 | pIRL_*rpoB*_MAB3869c_sg2_Top | BsmBI-Cut | GGGAAGGTGCCCATATCGGTCATCAT | pEAR55 |
| #P5 | pIRL_*rpoB*_MAB3869c_sg2_Bottom | BsmBI-Cut | AAACATGATGACCGATATGGGCACCT | pEAR55 |
| #P6 | pIRL_*mmpL4b*_MAB4115c_sg1_Top | BsmBI-Cut | GGGAGGGCAACCGGGTGAAGTGCAGGC | pEAR49 |
| #P7 | pIRL_*mmpL4b*_MAB4115c_sg1_Bottom | BsmBI-Cut | AAACGCCTGCACTTCACCCGGTTGCCC | pEAR49 |
| #P8 | pIRL_*mmpL4b*_MAB4115c_sg2_Top | BsmBI-Cut | GGGAGCGGTTGAACGGCAAGACCACTAC | pEAR50 |
| #P9 | pIRL_*mmpL4b*_MAB4115c_sg2_Bottom | BsmBI-Cut | AAACGTAGTGGTCTTGCCGTTCAACCGC | pEAR50 |
| #P10 | pIRL_*mmpL4b*_MAB4115c_sg3_Top | BsmBI-Cut | GGGAGTGTCCAGCGCTTTGCAGACTCAG | pEAR51 |
| #P11 | pIRL_*mmpL4b*_MAB4115c_sg3_Bottom | BsmBI-Cut | AAACCTGAGTCTGCAAAGCGCTGGACAC | pEAR51 |

**Table S2. List of the plasmids used in this study**

| **Name** | **Alternative Name** | **Short Description** | **Reference and/or Source** |
| --- | --- | --- | --- |
| pJL31 | pIRL117_*psmyc::mWasabi* | Kanamycin resistant L5-integrative vector for CRISPR interference in *M.smegmatis* with constitutive expression of mWasabi under the control of the psmyc promotor (psmyc::mWasabi) | [22] - Addgene Plasmid #227428 |
| pJL32 | pIRL117_*psmyc::dTomato* | Kanamycin resistant L5-integrative vector for CRISPR interference in *M.smegmatis* with the constitutive expression of dTomato under the control of the psmyc promotor (psmyc::dTomato) | [22] - Addgene Plasmid #227429 |
| pJL33 | pIRL2_*psmyc::mWasabi* | Kanamycin resistant L5-integrative vector for CRISPR interference in *M.tuberculosis* with the constitutive expression of mWasabi under the control of the psmyc promotor (psmyc::mWasabi) | [22] - Addgene Plasmid #227430 |
| pJL34 | pIRL2_*psmyc::dTomato* | Kanamycin resistant L5-integrative vector for CRISPR interference in *M.tuberculosis* with the constitutive expression of dTomato under the control of the psmyc promotor (psmyc::dTomato) | [22] - Addgene Plasmid #227431 |
| pJL35 | pJL32_MSMEG_*rpoB* | Kanamycin resistant L5-integrative vector for CRISPR interference of *rpoB* in *M.smegmatis* with the constitutive expression of dTomato under the control of the psmyc promotor (psmyc::dTomato) | [22] |
| pJL36 | pJL32_MSMEG_*mmpL3* | Kanamycin resistant L5-integrative vector for CRISPR interference of *mmpL3* in *M.smegmatis* with the constitutive expression of dTomato under the control of the psmyc promotor (psmyc::dTomato) | [22] |
| pJL37 | pJL32_MSMEG_*mmpL4b* | Kanamycin resistant L5-integrative vector for CRISPR interference of *mmpL4b* in *M.smegmatis* with the constitutive expression of dTomato under the control of the psmyc promotor (psmyc::dTomato) | [22] |
| pEAR46 | pJL34_MAB4508_*mmpL3* | Kanamycin resistant L5-integrative vector for CRISPR interference of *mmpL3* in *M.abscessus* with the constitutive expression of dTomato under the control of the psmyc promotor (psmyc::dTomato) | This study |
| pEAR49 | pJL34_MAB4115c_*mmpL4b_sg1* | Kanamycin resistant L5-integrative vector for CRISPR interference of *mmpL4b* in *M.abscessus* with the constitutive expression of dTomato under the control of the psmyc promotor (psmyc::dTomato) | This study |
| pEAR50 | pJL34_MAB4115c_*mmpL4b_sg2* | Kanamycin resistant L5-integrative vector for CRISPR interference of *mmpL4b* in *M.abscessus* with the constitutive expression of dTomato under the control of the psmyc promotor (psmyc::dTomato) | This study |
| pEAR51 | pJL34_MAB4115c_*mmpL4b_sg3* | Kanamycin resistant L5-integrative vector for CRISPR interference of *mmpL4b* in *M.abscessus* with the constitutive expression of dTomato under the control of the psmyc promotor (psmyc::dTomato) | This study |
| pEAR55 | pJL34_MAB3869c_*rpoB* | Kanamycin resistant L5-integrative vector for CRISPR interference of *rpoB* in *M.abscessus* with the constitutive expression of dTomato under the control of the psmyc promotor (psmyc::dTomato) | This study |
